# Positive Carrier for MAPT

**DOI:** 10.1002/alz.086681

**Published:** 2025-01-03

**Authors:** Linde L Jacobs, Cure MAPT FTD

**Affiliations:** ^1^ Cure MAPT FTD, Denver, CO USA

## Abstract

Dementia was a condition I was aware of from a very young age as I witnessed my grandmother decline, and my mother step into the role as a caregiver, health care director and power of attorney. I was taught the foundation for this process by direct observation of my mother’s actions. One aspect of caregiving that isn’t teachable is the emotional pain, anguish, sadness and guilt that often accompanies that role. My mother started exhibiting symptoms of FTD in 2010, right as I was graduating college. Despite having a family history, it took 8 harrowing years to receive a correct diagnosis and genetic testing. It was then confirmed that my family possesses the MAPT mutation causing bvFTD. My mother passed away in 2021 at the age of 62. Shortly after her passing, I learned that I was a positive carrier for the MAPT gene as well.

This started a new mission by using my past experience, to change the present, in hopes for a future. Currently, my future is not a guarantee, but I will not wait for the next 10 years hoping for a cure by staying quiet. Instead, I am using this time of waiting to use my voice and failed experiences with my mother, to motivate change, understanding and awareness for FTD. Further, I am using the knowledge of my genetic status to engage with the scientists and researchers to help in ways I am capable of driving progress forward. Being honest about the psychosocial implications genetic testing has on a person is that this test doesn’t have to render them useless, but use it to propel them forward. I look toward the future with hope knowing that the community fighting this disease is doing everything they can to find a solution. If there isn’t light at the end of my tunnel, I hope the light of the journey I led will burn so bright with persistence, strength and perseverance that my daughters will have the resilience to survive another generation of caregiving. All while they await their unpromised future because of genetic MAPT FTD.

